# Lamina Cribrosa Changes Following Pars Plana Vitrectomy and Silicone Oil Injection for Rhegmatogenous Retinal Detachment: A Contralateral Eye Study

**DOI:** 10.18502/jovr.v21.14209

**Published:** 2026-07-13

**Authors:** Homayoun Nikkhah, Fatemeh Foroudi Eshtahbanati, Sadid Hooshmandi, Saeed Karimi, Amir Keyvan Sazgar, Hosein Nouri, Seyed-Hossein Abtahi, Ali Forouhari

**Affiliations:** ^1^Ophthalmic Research Center, Research Institute for Ophthalmology and Vision Science, Shahid Beheshti University of Medical Sciences, Tehran, Iran; ^2^Clinical Research Development Unit, Torfeh Medical Center, Shahid Beheshti University of Medical Sciences, Tehran, Iran; ^3^School of Medicine, Tehran University of Medical Sciences, Tehran, Iran; ^4^School of Medicine, Isfahan University of Medical Sciences, Isfahan, Iran; ^5^These authors contributed equally to this work.

**Keywords:** Lamina Cribrosa, Optical Coherence Tomography, Retinal Detachment, Silicone Oil Tamponade, Vitrectomy

## Abstract

**Purpose:**

To assess changes in lamina cribrosa (LC) characteristics in eyes with silicone oil (SO) due to rhegmatogenous retinal detachment (RRD).

**Methods:**

This cross-sectional contralateral eye study was conducted on 49 patients with unilateral RRD who underwent pars plana vitrectomy and SO injection followed by SO removal. The contralateral eyes served as controls. The thickness and depth of LC in both eyes of the participants were evaluated using the enhanced depth imaging technique via optical coherence tomography.

**Results:**

The average age of the participants was 57.6 ± 10.5 years, and the mean duration of postoperative SO retention was 6.4 ± 1.7 months. The mean depth of LC was 347.6 ± 64.3 µm in eyes with SO tamponade and 329.6 ± 76.7 µm in healthy eyes (*P* = 0.232). On the other hand, the LC in eyes with SO tamponade was significantly thinner compared to that in healthy eyes (270.1 ± 45.1 µm vs 303.2 ± 48.6 µm; *P* < 0.0001). In addition, subgroup analysis revealed that similar findings were obtained after excluding patients who had received antiglaucoma medications to manage increased intraocular pressure (IOP).

**Conclusion:**

This study showed that eyes with SO injection exhibit a significantly thinner LC compared to healthy eyes, with no difference in the depth of the LC between the two groups.

##  INTRODUCTION

Silicone oil (SO) is widely used in vitreoretinal surgery as an intraocular tamponade agent. It provides benefits in repairing complex retinal detachments accompanied by conditions such as giant retinal tear, ocular trauma, or proliferative vitreoretinopathy (PVR).^[1,2]^ Previous studies have confirmed the efficacy and safety of SO as an endotamponade.^[3,4]^ One notable advantage of SO is its ability to serve as a long-term tamponade agent until anatomic retinal recovery is provided.^[5]^ However, a variety of complications, including cataract, secondary glaucoma, keratopathy, and SO-related vision loss (SORVL), have been associated with the use of SO, particularly in cases with longer-term tamponade.^[2]^ Optic nerve atrophy has also been reported following SO injection in some patients.^[6]^ SO is removed 3 to 6 months after pars plana vitrectomy (PPV) and achievement of retinal reattachment.

Although the effects of SO on various ocular structures, such as cornea, trabecular meshwork, retina, and optic nerve, have been studied, no specific study has investigated changes in the lamina cribrosa (LC) after SO injection.^[7]^ LC is a collagenous meshwork trabecula that is located in the posterior scleral canal of the optic nerve head (ONH).^[8]^ It surrounds and supports retinal ganglion cell (RGC) axons, the central retinal vein, and the central retinal artery.^[9]^

LC is a dynamic structure that responds to strain and acts as the interface between the high-pressure intraocular space and the low-pressure retrobulbar space.^[8,9]^ LC morphologic alterations (visible on *in vivo* imaging technologies) are reported in several ophthalmic pathologies, including intraocular hypertension, glaucomatous optic neuropathy, and myopia.^[8,10,11]^ LC thinning and posterior displacement are associated with optic neuropathy and subsequent visual impairment due to the disruption of axoplasmic flow within the optic nerve.^[12,13,14,15]^

In this study, our objective was to investigate changes in thickness and depth of LC in eyes that underwent PPV and SO tamponade followed by SO removal and compare the findings with their fellow healthy eyes.

##  METHODS

This cross-sectional contralateral eye study included patients who underwent surgical treatment for rhegmatogenous retinal detachment (RRD) at Torfeh Medical Center, a tertiary eye center, from December 2021 to February 2022. The institutional review board and the ethics committee of Shahid Beheshti University of Medical Sciences reviewed and approved the study protocol (IR.SBMU.MSP.REC.1401.454). This study was conducted in compliance with the ethical guidelines outlined in the Declaration of Helsinki. Written informed consent was obtained from all eligible patients prior to their participation in the study.

Patients were selected according to the following inclusion criteria: 18 years of age or older, history of PPV with SO tamponade for repair of primary RRD, and subsequent SO removal after retinal re-attachment. The exclusion criteria comprised of previous ocular surgery in either eye, high refractive error of ± 6.00 D, an axial length difference of > 0.3mm between the eyes, preoperative high intraocular pressure (IOP) or glaucoma, presence of other eye pathologies (including diabetic retinopathy, retinal vein occlusion, age-related macular degeneration, and optic nerve abnormalities), IOP > 21 mmHg that sustained for more than 72 hours throughout the entire postoperative period, and occurrence of any intraoperative or postoperative complications. A patient was also excluded if good-quality images (quality score > 20) could not be obtained using enhanced depth imaging optical coherence tomography (EDI-OCT) for more than five sections of the scan.

The same surgeon (HN) performed the operations on all patients diagnosed with RRD. In all cases, a standard 23-gauge PPV and SO tamponade insertion were performed. Following vitreous removal and shaving of the periphery, the detached retina was flattened using perfluorocarbon fluid, and endolaser photocoagulation was performed. After the air–fluid exchange, SO (either Oxane 5700 [Bausch & Lomb, Inc., Waterford, Ireland] or SIL-5000-S [DORC, Dutch Ophthalmic, USA]) was injected. The patients were instructed to remain prone for 2 to 4 weeks. The IOP of the operated eye was assessed regularly.

In addition to ocular history, all included patients underwent comprehensive ophthalmic examinations, including slit-lamp biomicroscopy, Goldmann applanation tonometry, and best-corrected visual acuity (BCVA) assessment with a Snellen chart. Indirect ophthalmoscopy was conducted with a 20-D lens to evaluate the status of the retina and SO emulsification. Visual acuity was presented as the logarithm of the minimum angle of resolution (LogMAR).

Axial length was measured with IOLMaster (Zeiss, Jena, Germany). The EDI-OCT was utilized to assess the thickness and depth of the LC. After pupil dilation, the spectral-domain OCT device (Spectralis HRA + OCT, Heidelberg Engineering, Heidelberg, Germany) was configured to obtain a rectangular 15º × 10º image centered on the optic disc in enhanced-depth imaging mode. The image was divided into approximately 65 raster horizontal sections, with each section averaging 100 OCT frames. EDI-OCT measurements were conducted by an experienced operator and were checked by a retinal specialist (HN).

The LC was identified as the area between the inner and outer lines of the more reflective region in the central horizontal section of the ONH. A line connecting the ends of the Bruch's membrane on each side of the B-scan—defined as Bruch's membrane opening—was considered as the reference line. In addition, the thickness and depth of the LC were assessed using the caliper tool in the Heidelberg Eye Explorer software. For measuring the parameters, three B-scan frames passing through the center, mid-inferior, and mid-superior regions of the ONH were selected, and the average was reported. Lamina cribrosa thickness (LCT) was measured as the perpendicular distance between the hyperreflective anterior and posterior surfaces of the LC. The lamina cribrosa depth (LCD) was reported as the average of three measurements that were made perpendicular to the reference line (positioned at the center and 100 µm nasal and temporal to the center of the reference line) and the anterior margin of the LC at the center of the ONH. The cup–disc ratio was measured by DRI OCT Triton (Topcon, Tokyo, Japan). The vertical cup–disc ratio was determined based on color fundus photographs.

The thickness and depth of the LC were the primary outcome variables in this study. Visual acuity was considered a secondary outcome. All measurements described above were performed 6 months after the operation and 1-7 days before SO removal on both eyes of the patients (the SO eye and the fellow control eye).

**Table 1:** Comparison of ocular parameters in eyes with and without silicone oil tamponade

	**Eyes with SO**	**Eyes without SO**	***P*** **-value**
LCT (µm)	270.7 ± 45.1	303.2 ± 48.6	< 0.001
LCD (µm)	347.6 ± 64.3	329.6 ± 76.7	0.232
Axial length (mm)	23.6 ± 1.3	23.7 ± 1.2	0.586
BCVA (LogMAR)	0.96 ± 0.32	0.17 ± 0.15	< 0.001
IOP (mmHg)	14.7 ± 2.7	12.1 ± 2.1	< 0.001
Cup-to-disc ratio	0.41 ± 0.14	0.33 ± 0.75	0.09
SO, silicon oil; LCD, lamina cribrosa thickness; LCD, lamina cribrosa depth; µm, micrometers, mm, millimeters; BCVA, best-corrected visual acuity; LogMAR, logarithm of the minimum angle of resolution; IOP, intraocular pressure. The values represent mean ± standard deviation.			

**Table 2:** Comparison of ocular parameters in eyes with and without silicone oil tamponade among patients who were not taking anti-glaucoma medication following surgery

	**Eyes with SO**	**Eyes without SO**	***P*** **-value**
LCT (µm)	269.5 ± 46.8	305.7 ± 50.2	0.01
LCD (µm)	340.2 ± 62.3	325.1 ± 75.1	0.41
Axial length (mm)	23.3 ± 1.3	23.5 ± 1.3	0.52
BCVA (LogMAR)	0.97 ± 0.33	0.18 ± 0.17	< 0.001
IOP (mmHg)	14.6 ± 2.6	12.3 ± 2.1	0.001
Cup-to-disc ratio	0.36 ± 0.11	0.34 ± 0.08	0.72
SO, silicon oil; LCD, lamina cribrosa thickness; LCD, lamina cribrosa depth; µm, micrometers; mm, millimeters; BCVA, best-corrected visual acuity; LogMAR, logarithm of the minimum angle of resolution; IOP, intraocular pressure. The values represent mean ± standard deviation.			

**Table 3:** Comparison of ocular parameters in operated eyes of individuals taking and not taking antiglaucoma medication following surgery

	**With antiglaucoma medication**	**Without antiglaucoma medication**	***P*** **-value**
LCT (µm)	250.3 ± 42.9	269.4 ± 46.8	0.21
LCD (µm)	361.0 ± 67.8	340.2 ± 62.3	0.3
Axial length (mm)	23.9 ± 1.1	23.3 ± 1.3	0.11
BCVA (LogMAR)	0.93 ± 0.3	0.97 ± 0.3	0.74
IOP (mmHg)	15.7 ± 2.5	14.6 ± 2.6	0.24
Cup-to-disc ratio	0.58 ± 0.08	0.36 ± 0.11	0.008
SO, silicon oil; LCD, lamina cribrosa thickness; LCD, lamina cribrosa depth; µm, micrometers; mm, millimeters; BCVA, best-corrected visual acuity; LogMAR, logarithm of the minimum angle of resolution; IOP, intraocular pressure. The values represent mean ± standard deviation.			

**Table 4:** Comparison of ocular parameters in nonoperated eyes of individuals taking and not taking antiglaucoma medication following surgery

	**With antiglaucoma medication**	**Without antiglaucoma medication**	***P*** **-value**
LCT (µm)	269.9 ± 46.9	305.7 ± 50.2	0.59
LCD (µm)	337.8 ± 81.4	325.1 ± 75.1	0.6
Axial length (mm)	23.9 ± 0.9	23.5 ± 1.3	0.32
BCVA (LogMAR)	0.15 ± 0.1	0.18 ± 0.2	0.54
IOP (mmHg)	12.2 ± 2	12.3 ± 2.1	0.93
Cup-to-disc ratio	0.31 ± 0.09	0.34 ± 0.08	0.46
SO, silicon oil; LCD, lamina cribrosa thickness; LCD, lamina cribrosa depth; µm, micrometers; mm, millimeters; BCVA, best-corrected visual acuity; LogMAR, logarithm of the minimum angle of resolution; IOP, intraocular pressure. The values represent mean ± standard deviation.			

### Statistical Analyses

The data were presented as mean ± SD for continuous variables and percentages for categorical variables. The distribution of quantitative variables was checked with the Kolmogorov–Smirnov test. The independent samples *t*-test and Mann–Whitney U test were used to assess the difference of variables between the study groups. The Pearson correlation coefficient was used to evaluate the correlation between LCT and the duration of SO tamponade, as well as the correlation between LCT and IOP. All statistical analyses were conducted in SPSS, version 26.0. (IBM Corp, Armonk, NY, USA). Statistical significance was considered at *P* < 0.05.

##  RESULTS

The study enrolled 49 patients who underwent PPV and SO injection for RRD management. The average age of the participants was 57.6 ± 10.5 years, with 27 (55%) males and 22 (44%) females. The mean duration of surgery was 41.9 ± 100.4 minutes. SO with a viscosity of 5700 centistokes was used for 38 eyes, while the remaining 11 eyes received SO with 5000 centistokes viscosity. The mean duration of SO retainment was 6.4 ± 1.7 months postoperatively.

The mean LCD was 347.6 ± 64.3 µm in eyes with previous SO tamponade and 329.6 ± 76.7 µm in healthy eyes (*P* = 0.232). On the other hand, as seen in Table 1, the LC in eyes with SO tamponade (270.1 ± 45.1 µm) was significantly thinner compared to that in healthy eyes (303.2 ± 48.6 µm; *P* < 0.0001). However, no significant correlation was observed between changes in LCT and the duration of SO tamponade (*r* = 0.157, *P* = 0.34).

The mean IOP in eyes with SO tamponade was higher (14.7 ± 2.7 mmHg) compared to the contralateral eyes (12.1 ± 2.1 mmHg; *P* < 0.001), although the difference may not be clinically significant. We did a correlation analysis, and there was no significant correlation between postoperative IOP and LCT (*r* = - 0.13, *P* = 0.4). Regarding visual acuity, the LogMAR BCVA in eyes with SO tamponade was 0.96 ± 0.32, while it was 0.17 ± 0.15 in healthy eyes (*P* < 0.001). No statistically significant difference was found in terms of mean axial length between eyes with SO tamponade and healthy eyes (*P* = 0.59) [Table 1].

Antiglaucoma medications (with an average of 0.8 ± 1.7 drugs) were prescribed in 19 eyes (38.8%) to manage the increased IOP following SO injection. After excluding eyes that received antiglaucoma medications, a comparative analysis was performed. According to the findings, the mean LCT in eyes treated with SO was measured at 269.5 ± 46.8 µm, whereas in healthy eyes it was 305.7 ± 50.2 µm (*P* = 0.01). Furthermore, the mean LCD in eyes treated with SO showed no significant difference compared to the value in the healthy eyes (*P* = 0.41). Additional variables and their corresponding values are presented in Table 2.

The comparison of evaluated parameters in eyes with SO between individuals who received antiglaucoma medications and those who did not revealed no significant difference in terms of any of the parameters [Table 3]. Similarly, no statistically significant differences were observed in any of the parameters in nonoperated eyes based on antiglaucoma medication status [Table 4].

##  DISCUSSION

The etiology of visual impairment following SO tamponade has been attributed to various factors, including thinning of inner retinal layers in the macular region, reduction in myelinated optic nerve fibers, and optic neuropathy due to granulomatous inflammatory reaction and infiltration of SO into the retrolaminar space.^[16,17,18,19,20,21]^ It is not clear whether optic neuropathy or macular changes are the primary cause of SORVL.^[22]^ While numerous studies have investigated the impact of SO tamponade on retinal layers and the migration of emulsified SO into retinal layers, none have specifically focused on changes in the LC. It has been reported that changes in LC structure could lead to axonal degeneration and apoptotic death of RGCs.^[23]^ Evaluating LC characteristics in eyes with SO tamponade could shed light on the optic-related pathologies which could lead to visual impairment.

The present study revealed a significant decrease in the LC thickness in eyes with SO tamponade compared to the contralateral healthy eyes. On the other hand, although eyes with SO showed a deeper LC compared to the fellow eyes, the observed difference in LCD was not significant.

The translaminar pressure gradient (TLPG) depends on IOP, retrolaminar tissue pressure, and LC thickness.^[24]^ Disturbance in TLPG can potentially damage structures within the LC, particularly RGC axons.^[25]^ Accordingly, changes in LCT—whether due to the SO itself or the subsequently increased IOP—can lead to a disturbance in TLPG and could be suggested as one of the factors associated with vision impairment following SO tamponade.

An increase in IOP can occur any time after SO tamponade, ranging from mild and transient to severe and persistent.^[26]^ Elevated IOP may also be related to factors such as undiagnosed glaucoma,^[27]^ inflammation due to surgery, postoperative steroid therapy, SO emulsification and migration into the anterior chamber causing trabecular meshwork obstruction, and angle closure secondary to the pupillary block.^[28,29,30,31,32,33]^ Early reports indicated a 20-50% increase in the rate of delayed elevated IOP and glaucoma in eyes with SO,^[34]^ but it has decreased to 3-30% in recent studies, possibly due to surgical and therapeutic advancements.^[35]^

In a study on patients with glaucoma conducted by Lee et al, it was reported that decreasing IOP after trabeculectomy led to a decrease in the depth of the LC and an increase in its thickness. The magnitude of these changes was more pronounced with a greater reduction in IOP.^[36]^ On the other hand, a study comparing LC thickness in healthy eyes and eyes with ocular hypertension suggested that the observed thicker LC in eyes with ocular hypertension could be a remodeling response to elevated IOP.^[37]^

To investigate the effect of IOP on the evaluated parameters, we also carried out subgroup analysis among patients who did not undergo any antiglaucoma medication. The results showed a significantly thinner LC in eyes with SO compared to eyes without SO. Besides, there was no significant difference in the mean LCD value between eyes with and without SO. There were no statistically significant differences in LC depth and thickness between eyes that developed glaucoma after surgery and eyes that did not. Given the routine IOP evaluation and the use of antiglaucoma medications to control elevated IOP, the majority of eyes had normal IOP during our study period. Although the average IOP in eyes with SO was slightly higher than in healthy eyes, the extent of its effect on the LC parameters may not be significant. This suggests that, unlike IOP variations within the normal range, there are other factors related to the inherent characteristics of SO and its subsequent toxic and inflammatory effects that could also explain the reduction in LCT.

Regarding the effect of changes in LC and consequently TLPG, it has been reported that a deeper and thinner LC (which increases TLPG) in eyes with glaucoma is associated with a thinner retinal nerve fiber layer (RNFL).^[14,38]^ Another study found a significant association between thinner LC and a faster rate of RNFL thinning in primary open-angle glaucoma.^[39]^

The effects of SO on retinal layers have been investigated in several studies. Similar to a survey by Geber et al,^[40]^ Jurišić et al^[22]^ concluded that the peripapillary RNFL thickness was greater in eyes with SO tamponade compared to the fellow control eyes. However, they mentioned that eyes with elevated IOP that underwent surgery had thinner peripapillary RNFL thickness compared to eyes with normal IOP.^[22]^ The authors attributed RNFL thickening to the infiltration of macrophages and giant cells secondary to the inflammatory reaction. On the other hand, Lee et al demonstrated a decrease in RNFL thickness in eyes with SO.^[41]^ Caramoy et al^[42]^ reported a subsequent reduction in the thickness of inner retinal layers (ganglion cell and inner plexiform layer) in eyes with SO tamponade compared to the fellow eyes. Also, Christensen and la Cour proposed the reduction in inner retinal thickness (indicating neuronal loss in the macular area) as a possible explanation for visual loss after SO use,^[18]^ which corresponds with findings reported by Tode et al.^[19]^

Retinal thinning following SO has been attributed to various underlying factors such as induced mechanical stress, retinal dehydration, and high IOP.^[43,44]^ SO can block the movement of fluid from the vitreous cavity into the retina and nerve fiber layer in prone and upright positions and, thus, induce retinal dehydration.^[43]^ The same mechanisms may be responsible for LC thinning after SO tamponade. Furthermore, disrupted ion exchange between the vitreous and retina and the absence of the appropriate buffering effect of vitreous fluid against potassium and hydrogen ions within the Müller cells lead to increased intraretinal potassium levels, which can result in the thinning of the inner layers and degeneration of the outer layers.^[45]^ Additionally, inflammatory cytokines induced by SO and light phototoxicity to the posterior pole could lead to macular dysfunction.^[22]^ This inflammatory state causes toxic effects on ganglion cells.^[46,47]^ SO can also affect synaptic spaces in the outer plexiform layer.^[48]^ Overall, further research is required to investigate microstructural damage to the retina and optic nerve resulting from mechanical stress or biochemical toxicity.

This study has several limitations, including the small sample size and the study design, which may restrict the generalizability of the findings. The cross-sectional design of the study provides only a snapshot of the variables at a single time point, without indicating baseline values before SO injection or subsequent changes after oil drainage. Additionally, all measurements were performed by a single observer. Although it helped maintain consistency, it prevented evaluation of inter-observer variability and could introduce the risk of observer bias.

In summary, this study found that eyes with SO exhibited a significantly thinner LC than the contralateral healthy eyes. However, no significant difference in the depth of the LC was found between the two groups. It is recommended to conduct prospective observational studies with larger sample sizes and long follow-up periods in order to further understand LC characteristics following SO insertion and evaluate possible causes of SORVL. Additionally, it would be beneficial to re-evaluate the attributes of the LC before SO removal and compare them with the corresponding measurements after SO removal to gain a more comprehensive understanding of the impact of SO on this structure.

##  Financial Support and Sponsorship

None.

##  Conflicts of Interest

None.

## References

[1] Cibis PA, Becker B, Okun E, Canaan S (1962). The use of liquid silicone in retinal detachment surgery. Arch Ophthalmol.

[2] Chen Y, Kearns VR, Zhou L, Sandinha T, Lam WC, Steel DH (2021). Silicone oil in vitreoretinal surgery: Indications, complications, new developments and alternative long-term tamponade agents. Acta Ophthalmol.

[3] Kurt RA, Kapran Z (2022). Heavy silicone oil as an endotamponade in recurrent or complicated retinal detachment and macular hole. Turk J Ophthalmol.

[4] Wang Y, Huang Z, Zheng D, Liu J, Huang D, Zheng J (2021). Adequate silicone oil tamponade by utilizing the space of anterior segment for complicated retinal detachment: Technique, efficacy, and safety. Asia Pac J Ophthalmol (Phila).

[5] Barca F, Caporossi T, Rizzo S (2014). Silicone oil: Different physical proprieties and clinical applications. Biomed Res Int.

[6] Lucke KH, Foerster MH, Laqua H (1987). Long-term results of vitrectomy and silicone oil in 500 cases of complicated retinal detachments. Am J Ophthalmol.

[7] Wickham L, Asaria RH, Alexander R, Luthert P, Charteris DG (2007). Immunopathology of intraocular silicone oil: Enucleated eyes. Br J Ophthalmol.

[8] Paulo A, Vaz PG, Andrade De Jesus D, Sánchez Brea L, Van Eijgen J, Cardoso J (2021). Optical coherence tomography imaging of the lamina cribrosa: Structural biomarkers in nonglaucomatous diseases. J Ophthalmol.

[9] Jonas JB, Berenshtein E, Holbach L (2003). Anatomic relationship between lamina cribrosa, intraocular space, and cerebrospinal fluid space. Invest Ophthalmol Vis Sci.

[10] Seo JH, Kim TW, Weinreb RN (2014). Lamina cribrosa depth in healthy eyes. Invest Ophthalmol Vis Sci.

[11] Han JC, Cho SH, Sohn DY, Kee C (2016). The characteristics of lamina cribrosa defects in myopic eyes with and without open-angle glaucoma. Invest Ophthalmol Vis Sci.

[12] Burgoyne CF, Downs JC, Bellezza AJ, Suh JK, Hart RT (2005). The optic nerve head as a biomechanical structure: A new paradigm for understanding the role of IOP-related stress and strain in the pathophysiology of glaucomatous optic nerve head damage. Prog Retin Eye Res.

[13] Lee EJ, Kim TW, Weinreb RN, Park KH, Kim SH, Kim DM (2011). Visualization of the lamina cribrosa using enhanced depth imaging spectral-domain optical coherence tomography. Am J Ophthalmol.

[14] Hao L, Xiao H, Gao X, Xu X, Liu X (2019). Measurement of structural parameters of the lamina cribrosa in primary open-angle glaucoma and chronic primary angle-closure glaucoma by optical coherence tomography and its correlations with ocular parameters. Ophthalmic Res.

[15] Tatham AJ, Miki A, Weinreb RN, Zangwill LM, Medeiros FA (2014). Defects of the lamina cribrosa in eyes with localized retinal nerve fiber layer loss. Ophthalmology.

[16] Budde M, Cursiefen C, Holbach LM, Naumann GO (2001). Silicone oil-associated optic nerve degeneration. Am J Ophthalmol.

[17] Mirza E, Şatırtav G, Oltulu R, Kerimoğlu H, Gündüz MK (2019). Subfoveal choroidal thickness change following pars plana vitrectomy with silicone oil endotamponade for rhegmatogenous retinal detachment. Int Ophthalmol.

[18] Christensen UC, la Cour M (2012). Visual loss after use of intraocular silicone oil associated with thinning of inner retinal layers. Acta Ophthalmol.

[19] Tode J, Purtskhvanidze K, Oppermann T, Hillenkamp J, Treumer F, Roider J (2016). Vision loss under silicone oil tamponade. Graefes Arch Clin Exp Ophthalmol.

[20] Papp A, Kiss EB, Tímár O, Szabó E, Berecki A, Tóth J (2007). Long-term exposure of the rabbit eye to silicone oil causes optic nerve atrophy. Brain Res Bull.

[21] Chikmah FA, Ichsan AM, Islam IC, Hendarto J, Muhiddin HS, Budu (2023). Retinal nerve fiber layer changes after intraocular silicone oil tamponade in rhegmatogenous retinal detachment. Vision (Basel).

[22] Jurišić D, Geber MZ, Ćavar I, Utrobičić DK (2018). Retinal layers measurements following silicone oil tamponade for retinal detachment surgery. Semin Ophthalmol.

[23] Yazdani S, Naderi Beni A, Pakravan M (2021). Laminar and prelaminar tissue characteristics of glaucomatous eyes using enhanced depth imaging OCT. Ophthalmol Glaucoma.

[24] Price DA, Harris A, Siesky B, Mathew S (2020). The influence of translaminar pressure gradient and intracranial pressure in glaucoma: A review. J Glaucoma.

[25] Morgan WH, Chauhan BC, Yu DY, Cringle SJ, Alder VA, House PH (2002). Optic disc movement with variations in intraocular and cerebrospinal fluid pressure. Invest Ophthalmol Vis Sci.

[26] Casswell AG, Gregor ZJ (1987). Silicone oil removal. II. Operative and postoperative complications. Br J Ophthalmol.

[27] Mangouritsas G, Mourtzoukos S, Portaliou DM, Georgopoulos VI, Dimopoulou A, Feretis E (2013). Glaucoma associated with the management of rhegmatogenous retinal detachment. Clin Ophthalmol.

[28] Armaly MF (1963). Effect of corticosteroids on intraocular pressure and fluid dynamics: I. The effect of dexamethasone* in the normal eye. Arch Ophthalmol.

[29] Siegfried CJ, Shui YB, Tian B, Nork TM, Heatley GA, Kaufman PL (2017). Effects of vitrectomy and lensectomy on older rhesus macaques: Oxygen distribution, antioxidant status, and aqueous humor dynamics. Invest Ophthalmol Vis Sci.

[30] Shaikh S, Egbert PR, Goldblum RS, Wieland MR (2000). Granulomatous local cell reaction to intravitreal silicone. Arch Ophthalmol.

[31] Betis F, Leguay JM, Gastaud P, Hofman P (2003). Multinucleated giant cells in periretinal silicone granulomas are associated with progressive proliferative vitreoretinopathy. Eur J Ophthalmol.

[32] Parmley VC, Barishak YR, Howes EL, Crawford JB (1986). Foreign-body giant cell reaction to liquid silicone. Am J Ophthalmol.

[33] Jackson TL, Thiagarajan M, Murthy R, Snead MP, Wong D, Williamson TH (2001). Pupil block glaucoma in phakic and pseudophakic patients after vitrectomy with silicone oil injection. Am J Ophthalmol.

[34] Antoun J, Azar G, Jabbour E, Kourie HR, Slim E, Schakal A (2016). Vitreoretinal surgery with silicone oil tamponade in primary uncomplicated rhegmatogenous retinal detachment. Retina.

[35] Al-Jazzaf AM, Netland PA, Charles S (2005). Incidence and management of elevated intraocular pressure after silicone oil injection. J Glaucoma.

[36] Lee EJ, Kim TW, Weinreb RN (2012). Reversal of lamina cribrosa displacement and thickness after trabeculectomy in glaucoma. Ophthalmology.

[37] Kim JA, Kim TW, Lee EJ, Girard MJ, Mari JM (2020). Comparison of lamina cribrosa morphology in eyes with ocular hypertension and normal-tension glaucoma. Invest Ophthalmol Vis Sci.

[38] Fortune B, Cull G, Reynaud J, Wang L, Burgoyne CF (2015). Relating retinal ganglion cell function and retinal nerve fiber layer (RNFL) retardance to progressive loss of RNFL thickness and optic nerve axons in experimental glaucoma. Invest Ophthalmol Vis Sci.

[39] Lee EJ, Kim TW, Kim M, Kim H (2015). Influence of lamina cribrosa thickness and depth on the rate of progressive retinal nerve fiber layer thinning. Ophthalmology.

[40] Zoric Geber M, Bencic G, Vatavuk Z, Ivekovic R, Friberg TR (2015). Retinal nerve fibre layer thickness measurements after successful retinal detachment repair with silicone oil endotamponade. Br J Ophthalmol.

[41] Lee YH, Lee JE, Shin YI, Lee KM, Jo YJ, Kim JY (2012). Longitudinal changes in retinal nerve fiber layer thickness after vitrectomy for rhegmatogenous retinal detachment. Invest Ophthalmol Vis Sci.

[42] Caramoy A, Droege KM, Kirchhof B, Fauser S (2014). Retinal layers measurements in healthy eyes and in eyes receiving silicone oil-based endotamponade. Acta Ophthalmol.

[43] Shimoda Y, Sano M, Hashimoto H, Yokota Y, Kishi S (2010). Restoration of photoreceptor outer segment after vitrectomy for retinal detachment. Am J Ophthalmol.

[44] Benson SE, Schlottmann PG, Bunce C, Xing W, Charteris DG (2006). Optical coherence tomography analysis of the macula after vitrectomy surgery for retinal detachment. Ophthalmology.

[45] Winter M, Eberhardt W, Scholz C, Reichenbach A (2000). Failure of potassium siphoning by Müller cells: A new hypothesis of perfluorocarbon liquid-induced retinopathy. Invest Ophthalmol Vis Sci.

[46] Haider A, Bababeygy S, Lu S (2015). Cystoid macular edema following silicone oil tamponade for retinal detachment surgery. J Clin Ophthalmol.

[47] Ferrara M, Coco G, Sorrentino T, Jasani KM, Moussa G, Morescalchi F (2022). Retinal and corneal changes associated with intraocular silicone oil tamponade. J Clin Med.

[48] Newsom RS, Johnston R, Sullivan PM, Aylward GB, Holder GE, Gregor ZJ (2004). Sudden visual loss after removal of silicone oil. Retina.

